# Prospective observational study of nutritional status and oral supplement utilization in users of an elderly daycare service, employing a web-based Mini Nutritional Assessment Form (MNA plus)

**DOI:** 10.3389/fnut.2024.1375592

**Published:** 2024-03-05

**Authors:** Hiroki Takano, Yukikazu Kamada, Masaki Ichikawa, Sadao Yoshida

**Affiliations:** ^1^Nestlé Health Science Company, Nestlé Japan Ltd., Tokyo, Japan; ^2^Department of Rehabilitation, Chuzan Hospital, Okinawa, Japan; ^3^Department of Health and Nutrition, Okinawa University, Okinawa, Japan; ^4^Faculty of Health Sciences, Kinjo University, Hakusan, Ishikawa, Japan

**Keywords:** elderly daycare service, oral nutritional supplement, Mini Nutritional Assessment Form, sarcopenia, frailty

## Abstract

**Introduction:**

Seniors are vulnerable to frailty, a condition linked to falls, fractures, hospitalizations, and sarcopenia. Even with regular meals, senior daycare users are at risk for malnutrition.

**Methods:**

This study assessed malnutrition risk in daycare users, using the web-based Mini Nutritional Assessment Form (MNA®-SF). Individuals identified as malnourished or at risk were examined for changes in nutritional status with and without oral nutritional supplementation (ONS).

**Results:**

Of 507 subjects, 138 (27.2%) were malnourished or at risk. Discontinuation rates were 20.0% (7/35) for the ONS group and 40.0% (10/25) for the regular care (RC) group. Among 29 patients with measurable weight change after six months, 19 (ONS group) and 10 (RC group) participated. The ONS group exhibited significant increases in body weight (+1.4 ± 2.9 kg, *p* < 0.01), body mass index (BMI) (+0.6 ± 0.9 kg/m^2^, *p* < 0.01), calf circumference (+3.2 ± 0.2 cm, *p* < 0.01), and grip strength (+1.2 ± 1.9 kg, *p* = 0.069). Conversely, the RC group showed no significant increases in body weight (+1.0 ± 1.9 kg, *p* = 0.146), BMI (+0.4 ± 0.8 kg/m^2^, *p* = 0.176), or grip strength (−0.7 ± 1.7 kg, *p* = 0.327), with decreased grip strength and calf circumference (−0.8 ± 0.9 cm, *p* < 0.05). In the ONS group, 52.6% (10/19) consumed over 400 kcal/day of ONS, and 84.2% maintained this intake for three months. Malnutrition is prevalent among daycare users.

**Conclusion:**

ONS influences weight, BMI, and calf circumference, potentially reducing discontinuation rates.

**Clinical trial registration:**

https://center6.umin.ac.jp/cgi-open-bin/ctr_e/ctr_view.cgi?recptno=R000049767, UMIN000043580.

## 1 Introduction

Global aging raises serious concerns. With an estimated 761 million individuals over 65 in 2021, and projections of nearly doubling to 1.6 billion by 2050, this demo-graphic shift demands attention (1). Japan, with 28.4% of its population aged 65 years or older (37 million individuals), stands at the forefront of this global phenomenon (2). In response to the challenges of a super-aging society, experts suggest that appropriate nutritional care, including adequate protein and energy intake, and optimal vitamin D levels, could play a crucial role in mitigating diverse healthcare issues (3). Elderly individuals are particularly vulnerable to undernutrition, with protein-energy malnutrition being especially prevalent among those requiring nursing care (4). Data from the 2019 National Health and Nutrition Survey by the Ministry of Health, Labor and Welfare reveals that 12.8% of men and 21.1% of women aged 65 years and older suffer from malnutrition (5). This chronic condition not only elevates the risk of cognitive decline, but also diminishes survival rates (6). Daycare services aim to address these concerns by reducing isolation in homebound individuals, maintaining physical and mental function, and alleviating the caregiving burden on families, ultimately enabling users to regain independent daily living at home (7). Unfortunately, reports suggest that nearly 60% of elderly daycare users experience malnutrition (8). Recent research efforts have focused on improving the nutritional status of older adults. One randomized controlled trial involving elderly patients undergoing rehabilitation for skeletal muscle compared an exercise-only group to a group receiving both exercise and nutritional supplements. The latter group demonstrated significant improvements in calf and arm circumferences, highlighting the significance of addressing malnutrition (9). However, a crucial gap remains: no studies have yet evaluated the impact of oral supplementation on nutritional status changes in elderly daycare users. Herein, we utilized the Mini Nutrition Assessment Short-Form (MNA®-SF) web form (10), to assess the nutritional status of daycare users. Furthermore, we investigated the effects of oral nutritional supplementation (ONS) on changes in nutritional status, sarcopenia, and overall prognosis, comparing the outcomes between groups receiving ONS and those not receiving it.

## 2 Materials and methods

The participants were users of 13 Solasto Corporation daycare facilities. From March 2021 to November 2022, they implemented a nutritional assessment using the MNA Plus. MNA Plus is a web-based version of MNA®-SF (developed by Mediaid Corporation, Tokyo, Japan, and sponsored by Nestlé Health Science Company, Tokyo, Japan).

### 2.1 Ethics

This prospective, multicenter study was conducted in compliance with the Declaration of Helsinki (revised in 2013), the Japanese Ethical Guidelines for Medical and Health Research Involving Human Subjects. This study was approved by the Ethical Review Committee of the Yamauchi Clinic, Rikeikai Medical Corporation, Tokyo, Japan (committee number: 20000007; approval date: March 18, 2021; UMIN Study ID: UMIN000043580).

### 2.2 Inclusion criteria

- Age 65 years or older at the time of consent.
- Identified by MNA Plus as being malnourished or at risk of malnutrition.

### 2.3 Exclusion criteria

- Using dietary supplements more than once a week at the time of consent.
- Having a fracture or other significant musculoskeletal impairment at the time of consent.
- Having difficulty weighing and measuring in daycare facilities.
- Having severe dementia.
- Having undergone surgery four weeks prior to the start of the observation period or being scheduled to undergo surgery during the observation period.
- Deemed by the investigator to be an unsuitable subject for this study.

### 2.4 Study procedure

**Figure 1:**
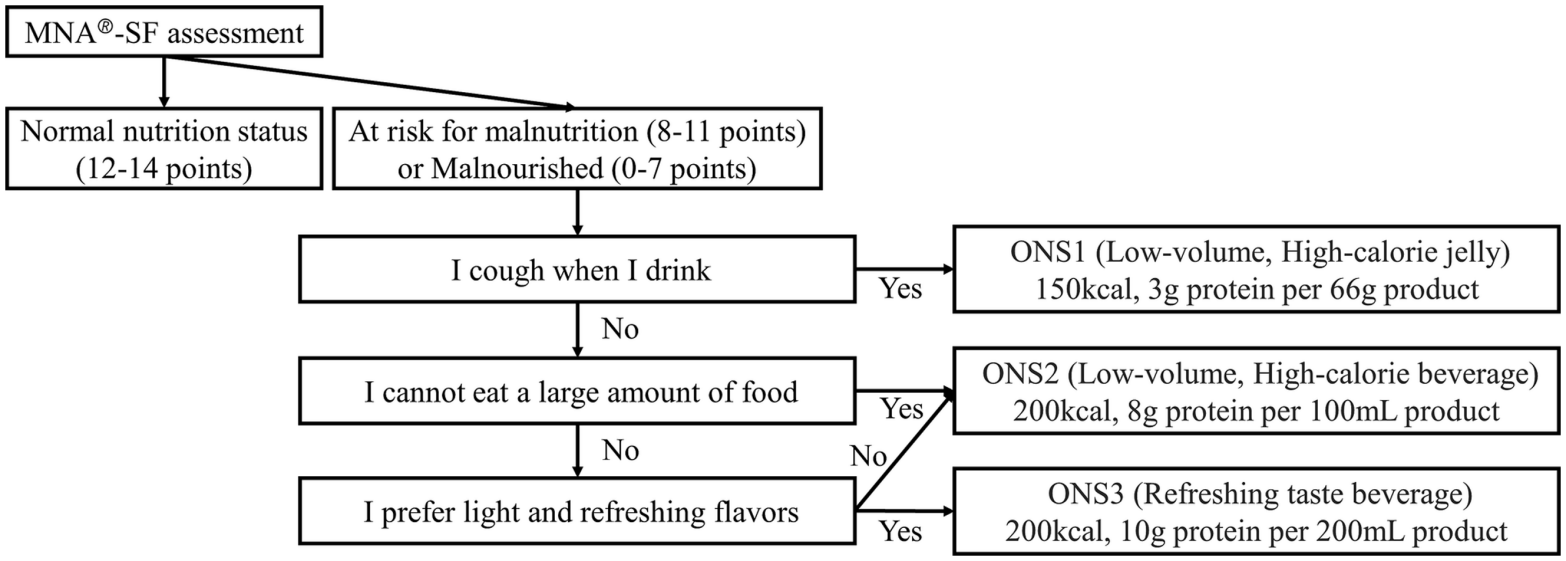
The evaluation algorithm of the mini nutrition assessment short-form plus. ONS1, Isocal® Jelly High Calorie; ONS2, Isocal® 100; ONS3, Isocal® Clear.

During the study period from March 2021 to November 2022, seven facilities pro-vided users with suggestions for ONS intake based on their MNA Plus assessments, in addition to their regular daycare services such as exercise, recreation, and lunch (ONS group). Six facilities provided users with regular daycare services only (RC group). The MNA Plus recommends the most appropriate ONS for each subject by considering not only nutritional status but also swallowing ease, amount of food per meal, and preferences. The ONS for each subject was selected from either ONS1 (product name: Isocal® Jelly High Calorie), ONS2 (product name: Isocal® 100), or ONS3 (product name: Isocal® Clear) and administered in doses of 300 kcal/day, 400 kcal/day, or 600 kcal/day based on the monthly MNA Plus assessment. Each ONS was formulated to match the subjects’ meal size, swallowing condition, preferences, and other factors (Figure 1). Compositions of ONS1, 2, and 3 are shown in Table 1. The decision to consume the suggested ONS was made at the subject’s or family member’s discretion.

**Table 1:** Compositions of ONS1, ONS2, and ONS3.

	ONS1	ONS2	ONS3
Product name	Isocal® Jelly High Calorie	Isocal® 100	Isocal® Clear
Shape	Jelly	Drink	Drink
Volume	66 g	100 mL	200 mL
Calories (kcal)	150	200	200
Protein (g)	3	8	10
Fat (g)	7.9	8	0
Carbohydrates (g)	16.8	25	40
Water (g)	38	70	166
Number of different vitamins	0	13	0
Number of different minerals	2	13	1

Values listed are per product.

### 2.5 Measurements

The primary endpoint was the change in body weight at three and six months from baseline.

Secondary endpoints were changes in body mass index (BMI), grip strength, and calf circumference at three and six months from baseline, and the percentage of subjects who continued taking ONS at six months from baseline.

### 2.6 Data analysis

All statistical analyses were performed using EZR (11), a statistical software package that extends the capabilities of R and R Commanders. Parametric data were expressed as mean ± standard deviation. Comparisons of means between the groups were performed using Welch’s *t*-test, comparisons of categorical variables were per-formed using Fisher’s exact test, and before/after comparisons were performed using the appropriate *t*-test. The significance level was set at *p* < 0.05.

## 3 Results

### 3.1 Nutritional assessment

**Figure 2:**
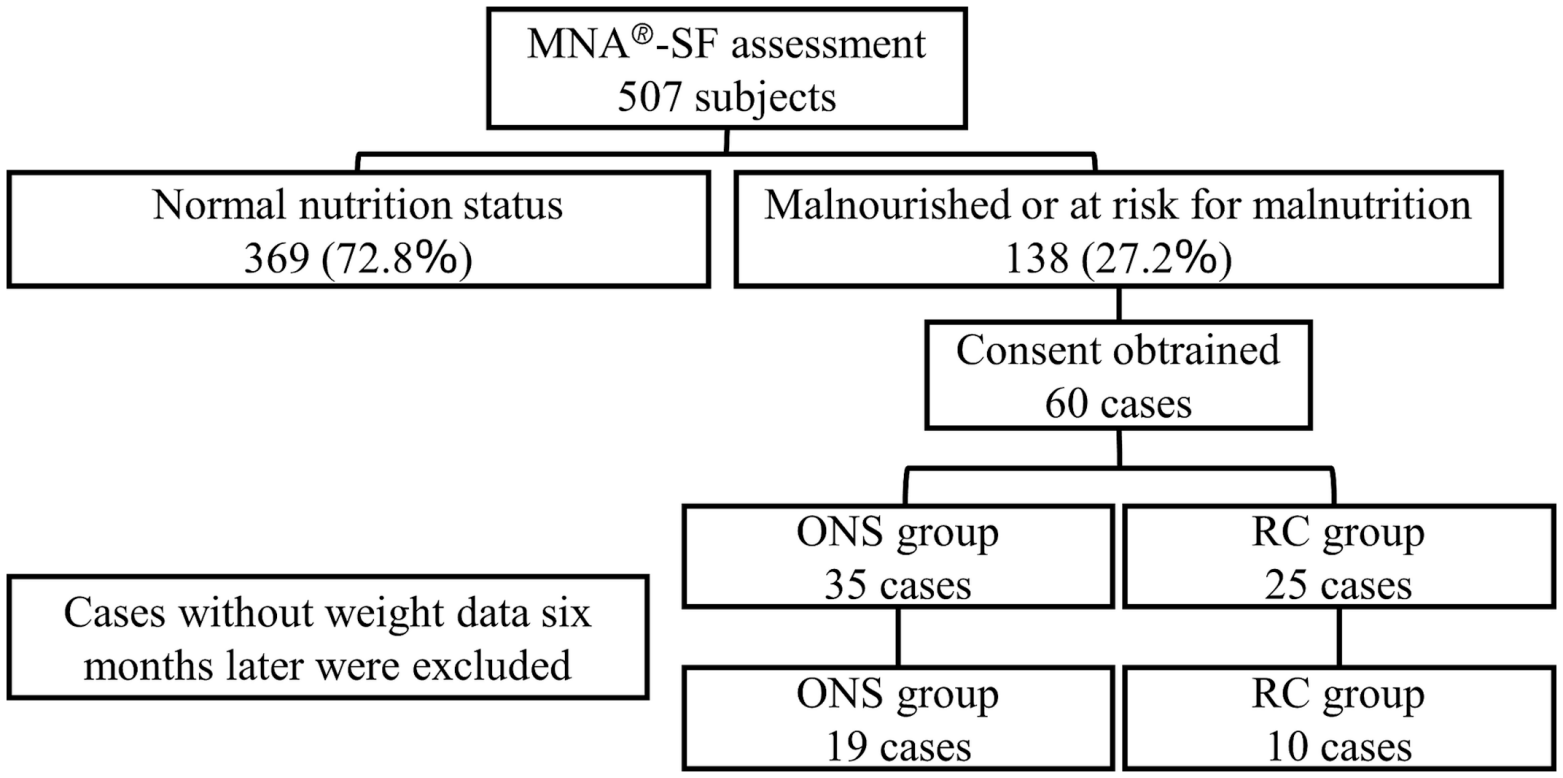
Case entry flow of oral nutritional supplementation (ONS) group and regular care (RC) group.

**Figure 3:**
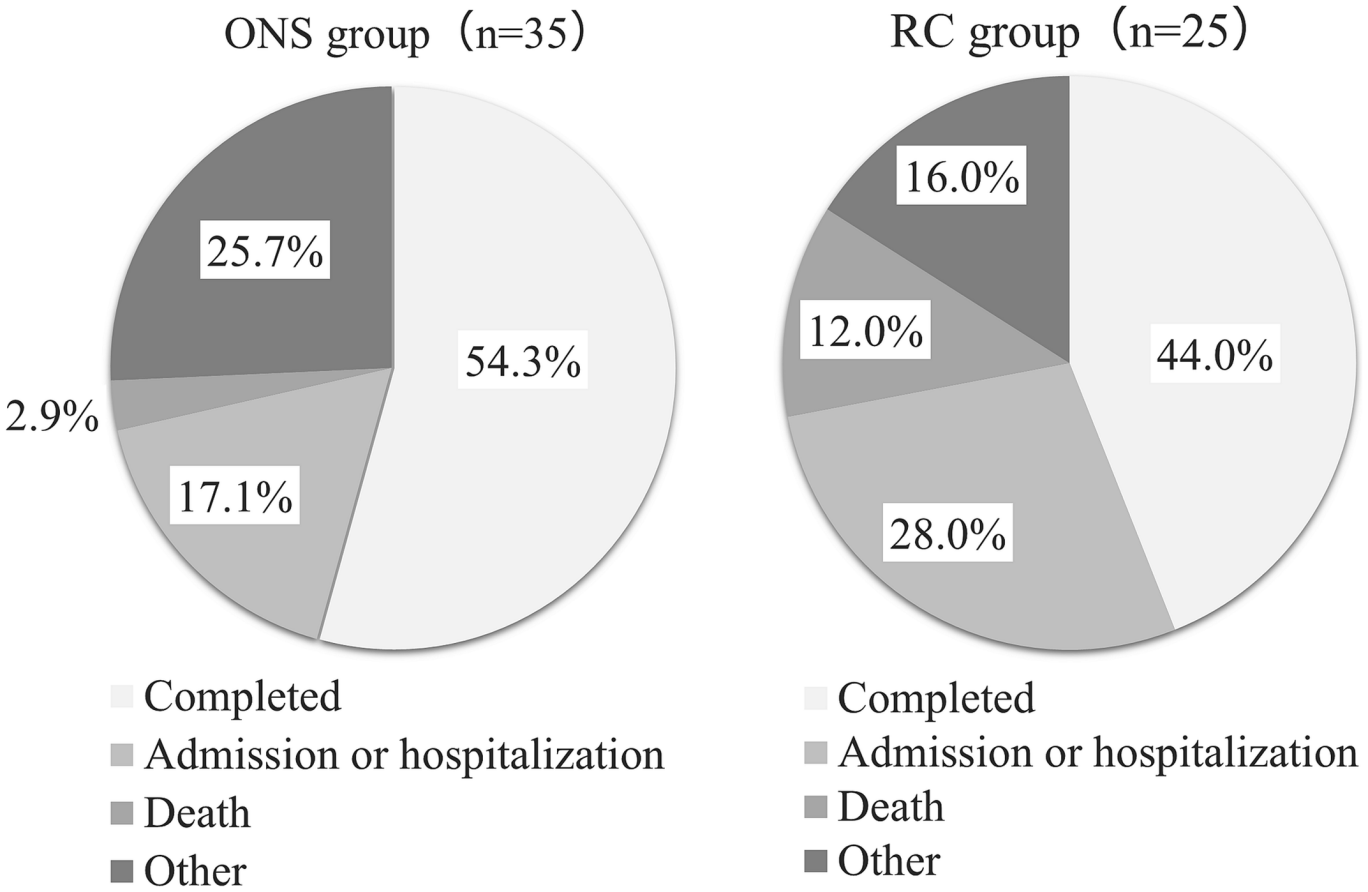
Outcomes of the ONS and RC groups. Other ONS group: family opposition (2), significant decrease in food intake (2), refusal to consume (2), stable meal intake at home, physician’s instructions, Intake of other ONS. Other of the RC group: withdrawal of consent (2), family opposition, and dietitian instructions.

The case entry flow is illustrated in Figure 2. Informed consent was obtained from all 60 patients. The outcomes for the 60 patients are shown in Figure 3. The rate of dis-continuation in service use due to patient deterioration (nursing home admission, hospitalization, and death) was 20.0% (7/35) in the ONS group and 40.0% (10/25) in the RC group. In the ONS group, 7 of the 19 patients who completed the observation voluntarily discontinued ONS before the end of the study because of improvement in malnutrition. Of the 60 cases, 29 were included in the analysis of weight, grip strength, etc., for which weight data were measured for up to six months, which was the primary endpoint.

### 3.2 Backgrounds

The backgrounds of these cases are listed in Table 2. Due to missing data, 11 cases in the ONS group and eight cases in the RC group were included in the analysis population for grip strength. Additionally, three cases in the ONS group and 10 cases in the RC group were included in the analysis population for calf circumference. Furthermore, 17 cases in the ONS group and nine cases in the RC group were included in the analysis population for instrumental activities for daily living.

**Table 2:** Backgrounds of oral nutritional supplementation (ONS) group and regular care (RC) group.

	ONS group (*n* = 19)	RC group (*n* = 10)	*p*-value
Sex (male/female)	7/12	4/6	0.876
Age	84.7 ± 5.6	82.5 ± 8.0	0.463
Body weight (kg)	47.6 ± 9.0	42.8 ± 9.0	0.211
BMI (kg/m^2^)	21.0 ± 3.0	16.9 ± 3.2	<0.01
Grip strength (kg)	13.2 ± 3.6 (*n* = 11)	18.5 ± 5.1 (*n* = 8)	<0.05
Calf circumference (cm)	26.3 ± 2.5 (*n* = 3)	29.9 ± 3.5 (*n* = 10)	0.171
MNA score	8.6 ± 2.1	8.6 ± 1.6	0.977
Malnourished (%)	26.3	30	0.844
ADL (proportion requiring assistance)			
Living, transferring, and moving	42.1% (8/19)	70.0% (7/10)	0.245
Meal	68.4% (13/19)	30.0% (3/10)	0.064
Discharge	10.5% (2/19)	40.0% (4/10)	0.143
Dressing	26.3% (5/19)	50.0% (5/10)	0.244
IADL	94.1% (16/17)	88.9% (8/9)	1

Ability to perform activities of daily living was compared between the groups using Fisher’s exact test. All other measures were compared between the groups using Welch’s *t*-test. BMI, Body Mass Index; MNA, Mini Nutritional Assessment; ADL, Activities of Daily Living; IADL, Instrumental Activities of Daily Living.

### 3.3 Changes in each parameter

**Figure 4:**
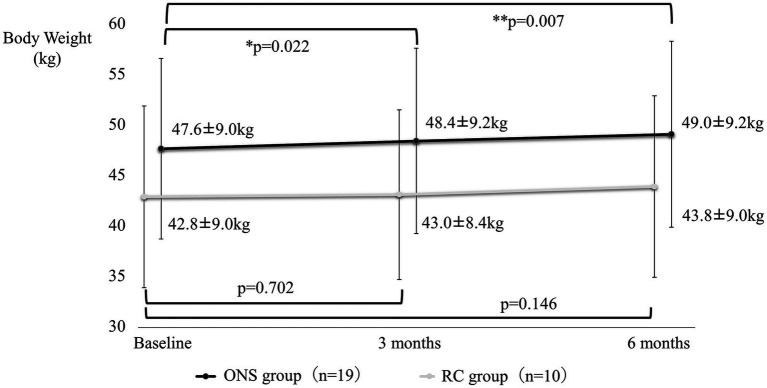
Change in body weight from baseline to 6 months. Mean ± standard deviation (SD); *p*-value: level of significance 5%, comparison before and after two-sided *t*-test.

**Figure 5:**
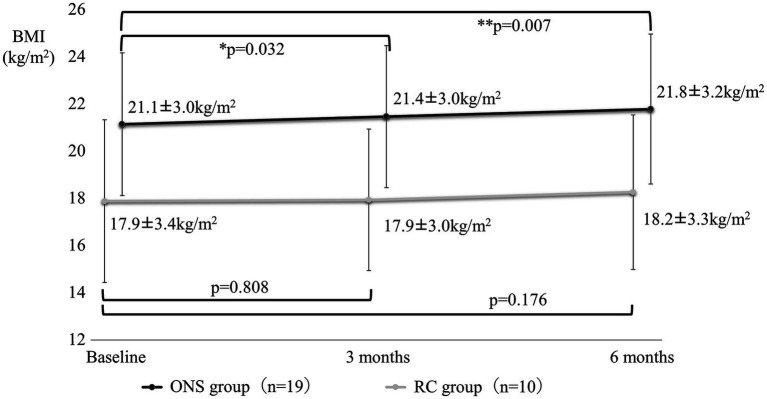
Change in body mass index from baseline to 6 months. Mean ± SD; *p*-value: level of significance 5%, comparison before and after two-sided *t*-test.

**Figure 6:**
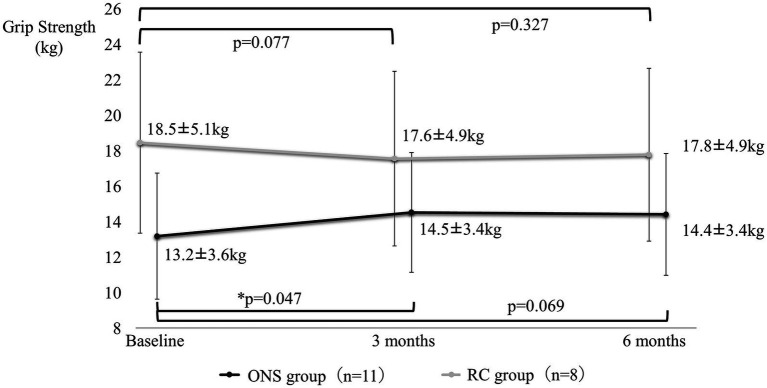
Change in grip strength from baseline to 6 months. Mean ± SD; *p*-value: level of significance 5%, comparison before and after two-sided *t*-test.

**Figure 7:**
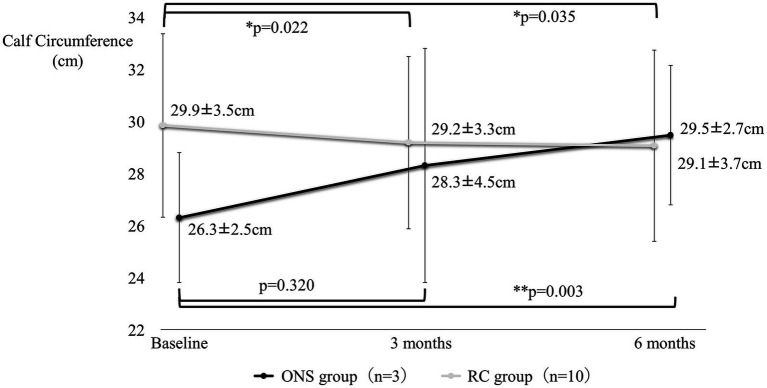
Change in calf circumference from baseline to 6 months. Mean ± SD; *p*-value: level of significance 5%, comparison before and after two-sided *t*-test.

Figures 4–7 show the changes in each item from baseline to six months. In the ONS group, weight and BMI increased significantly at three and six months, and grip strength increased significantly at three months. In the RC group, there were no significant differences in weight or BMI at three or six months, but grip strength tended to decrease. Calf circumference increased significantly in the ONS group at six months. In the RC group, calf circumference decreased significantly at three and six months, respectively.

### 3.4 Rate of continued ONS intake

In the ONS group, 52.6% (10/19) were recommended to consume >400 kcal/day of ONS. The rate of continued ONS intake in the third month was 84.2%. Among the patients who were recommended to consume 300 kcal/day of ONS, many improved their nutritional status after three months of ONS intake and many did not consume ONS after the fourth month.

### 3.5 Subgroup analysis

Most patients (73.7%, 14/19) consumed only ONS2 (ONS2 group). Of these, 42.9% (6/14 cases) were recommended to consume more than 400 kcal/day of ONS2, and all six patients continued to consume ONS2 for six months. The background and changes in weight, BMI, and grip strength in the ONS2 group are shown in Tables 3, 4. In the ONS2 group, there were no cases where the calf circumference could be measured, so it was not included in the table.

**Table 3:** Backgrounds of the ONS2 and RC groups.

	ONS2 group (*n* = 14)	RC group (*n* = 10)	*p*-value
Sex (male/female)	6/8	4/6	0.895
Age	83.4 ± 5.6	82.5 ± 8.0	0.784
Body weight (kg)	48.9 ± 9.2	42.8 ± 9.0	0.138
BMI (kg/m^2^)	21.7 ± 2.8	16.9 ± 3.2	<0.05
Grip strength (kg)	12.9 ± 3.8 (*n* = 8)	18.5 ± 5.1 (*n* = 8)	<0.05
MNA score	9.0 ± 1.9	8.6 ± 1.6	0.59
Malnourished (%)	21.4	30	0.658
ADL (proportion requiring assistance)			
Living, transferring, and moving	50.0% (7/14)	70.0% (7/10)	0.421
Meal	71.4% (10/14)	30.0% (3/10)	0.1
Discharge	14.3% (2/14)	40.0% (4/10)	0.192
Dressing	35.7% (5/14)	50.0% (5/10)	0.678
IADL	92.9% (13/14)	88.9% (8/9)	0.55

Ability to perform activities of daily living was compared between the groups using Fisher’s exact test. All other measures were compared between the groups using Welch’s *t*-test. BMI, Body Mass Index; MNA, Mini Nutritional Assessment; ADL, Activities of Daily Living; IADL, Instrumental Activities of Daily Living.

**Table 4:** Changes in each item in the ONS2 group.

	*n*	Baseline	3 months	*p*-value	6 months	*p*-value
Body weight (kg)	14	48.9 ± 9.2	49.8 ± 9.3	< 0.05	50.4 ± 9.6	< 0.05
BMI (kg/m^2^)	14	21.7 ± 2.8	22.1 ± 2.7	< 0.05	22.4 ± 3.0	< 0.01
Grip strength (kg)	8	12.9 ± 3.8	15.3 ± 3.6	< 0.001	15.0 ± 3.7	< 0.01

*p*-value: level of significance, 5%; comparison before and after two-sided *t*-test. BMI, Body Mass Index.

## 4 Discussion

Of the 507 subjects who underwent nutritional assessment using the MNA®-SF in the study’s daycare facilities, 138 (27.2%) were found to be malnourished or at risk of malnutrition. While previous reports found higher rates using the MNA®-SF (68 and 60% in community-dwelling elderly (7, 12)), this study using the MNA Plus showed a lower proportion likely due to its simplified assessment, allowing the inclusion of subjects with good nutritional status. This highlights the importance of regular nutritional assessments and interventions in day care facilities, as malnutrition often goes unnoticed. Day care services aim to maintain users’ physical and mental functions, reduce family caregiving burden, and enable independent home living. These facilities assist with activities of daily living, like eating and bathing, and training to maintain physical and oral cavity function (8). This study found that continuous ONS intake significantly increased grip strength. Sarcopenia (13, 14) and frailty progression (15) in the elderly are linked to a higher risk of various diseases, including cardiovascular (16, 17) and chronic respiratory ailments (18), diabetes (19–22), chronic kidney disease (23–25), dementia (20, 26), falls (27), and fractures (28). Our findings suggest that nutritional assessment with the MNA Plus and oral supplementation may help prevent body weight loss and declines in grip strength and calf circumference, key indicators of sarcopenia and frailty. The ONS group had an impressively high continuation rate of 84.2% at three months. This success likely stems from the MNA Plus’ personalized approach, selecting ONS based on each user’s nutritional needs, swallowing ease, portion size, and preferences. Notably, the low-volume, high-calorie ONS2 (100 mL/bottle) proved easy to consume and is believed to have contributed to continued use and improved nutritional status among malnourished users. Additionally, regular MNA Plus assessments encourage even those less interested in nutrition to actively track their status. This awareness, combined with ONS support, may have helped the RC group maintain their weight. The study suggests that continuous ONS intake in day care facilities can reduce disruptions that lead to nursing home admission, hospitalization, or death. Early nutritional assessment and sustained ONS use could pave the way for a robust day care system, ultimately enhancing users’ quality of life through service continuity, increased independence, and improved psychological well-being. Several limitations required consideration. First, the number of consenting participants was limited due to challenges in obtaining family consent, impacting the statistical power of the study. Second, the differing sizes and backgrounds of the participant groups must be factored in. Finally, missing data further reduced the available cases for analysis, potentially affecting the data’s completeness and generalizability. These limitations may have impacted the results’ reliability and applicability. Future studies should endeavor to improve case ascertainment, perhaps by creating a more accessible environment for families, and address these limitations to strengthen the validity of the findings.

## 5 Conclusion

Daycare users exhibited high malnourishment incidence and risk. Recommending ONS intake appears to increase body weight, BMI, and calf circumference while reducing the risk of nursing home admission, hospitalization, and death.

## Data availability statement

The raw data supporting the conclusions of this article will be made available by the authors, without undue reservation.

## Ethics statement

The studies involving humans were approved by the Ethical Review Committee of the Yamauchi Clinic, Rikeikai Medical Corporation, Tokyo, Japan. The studies were conducted in accordance with the local legislation and institutional requirements. The participants provided their written informed consent to participate in this study.

## Author contributions

HT: Conceptualization, Formal analysis, Funding acquisition, Writing – original draft, Writing – review & editing. YK: Project administration, Writing – review & editing. MI: Writing – review & editing. SY: Conceptualization, Methodology, Supervision, Validation, Writing – original draft, Writing – review & editing.
